# Tetra­guanidinium bis­[citrato(3−)]cuprate(II) dihydrate

**DOI:** 10.1107/S1600536809046170

**Published:** 2009-11-07

**Authors:** Mohammad T. M. Al-Dajani, Hassan H. Abdallah, Nornisah Mohamed, Chin Sing Yeap, Hoong-Kun Fun

**Affiliations:** a School of Pharmaceutical Sciences, Universiti Sains Malaysia, 11800 USM, Penang, Malaysia; b School of Chemical Sciences, Universiti Sains Malaysia, 11800 USM, Penang, Malaysia; c X-ray Crystallography Unit, School of Physics, Universiti Sains Malaysia, 11800 USM, Penang, Malaysia

## Abstract

The asymmetric unit of the title compound, (CH_6_N_3_)_4_[Cu(C_6_H_5_O_7_)_2_]·2H_2_O, contains one-half of a centrosymmetric Cu^II^ complex anion, two guanidinium cations and a water mol­ecule. The Cu^II^ ion, lying on a crystallographic inversion center, is hexa­coordinated with two citrate anions in a distorted octahedral geometry. An intra­molecular O—H⋯O hydrogen bond generates an *S*(6) ring motif. In the crystal structure, mol­ecules are linked into a three-dimensional framework by inter­molecular N—H⋯O and O—H⋯O hydrogen bonds.

## Related literature

For general background to citric acid and guanidine, see: Raczyńska *et al.* (2003 ▶); Yamada *et al.* (2009 ▶); Sigman *et al.* (1993 ▶). For a related structure with a guanidinium cation, see: Al-Dajani *et al.* (2009 ▶). For hydrogen-bond motifs, see: Bernstein *et al.* (1995 ▶). For the stability of the temperature controller used for the data collection, see: Cosier & Glazer (1986 ▶).
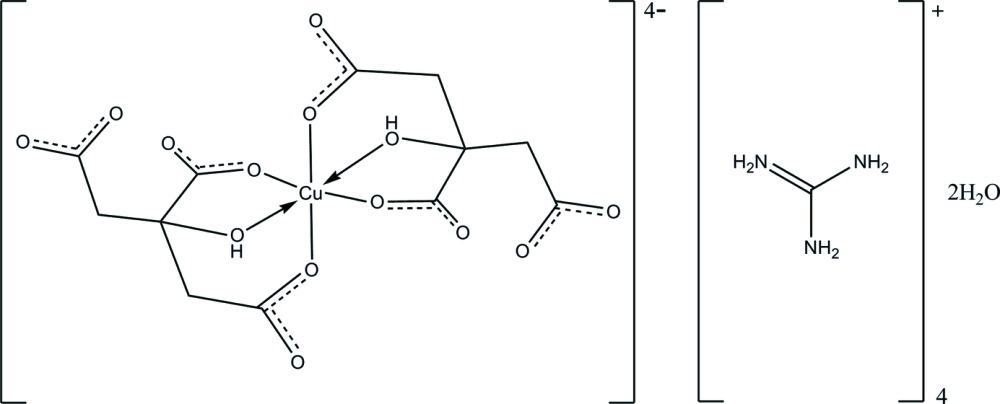


## Experimental

### 

#### Crystal data

(CH_6_N_3_)_4_[Cu(C_6_H_5_O_7_)_2_]·2H_2_O
*M* *_r_* = 718.12
Triclinic, 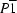

*a* = 9.0426 (1) Å
*b* = 9.7763 (2) Å
*c* = 10.3366 (2) Å
α = 96.503 (1)°
β = 105.441 (1)°
γ = 112.306 (1)°
*V* = 791.01 (2) Å^3^
*Z* = 1
Mo *K*α radiation
μ = 0.78 mm^−1^
*T* = 296 K
0.60 × 0.39 × 0.32 mm

#### Data collection

Bruker SMART APEXII CCD area-detector diffractometer
Absorption correction: multi-scan (**SADABS**; Bruker, 2005 ▶) *T*_min_ = 0.653, *T*_max_ = 0.787
37237 measured reflections
7051 independent reflections
6306 reflections with *I* > 2σ(*I*)
*R*_int_ = 0.024

#### Refinement

*R*[*F* ^2^ > 2σ(*F* ^2^)] = 0.031
*wR*(*F* ^2^) = 0.094
*S* = 1.05
7051 reflections
206 parameters
H-atom parameters constrained
Δρ_max_ = 0.44 e Å^−3^
Δρ_min_ = −0.49 e Å^−3^

### 

Data collection: *APEX2* (Bruker, 2005 ▶); cell refinement: *SAINT* (Bruker, 2005 ▶); data reduction: *SAINT*; program(s) used to solve structure: *SHELXTL* (Sheldrick, 2008 ▶); program(s) used to refine structure: *SHELXTL*; molecular graphics: *SHELXTL*; software used to prepare material for publication: *SHELXTL* and *PLATON* (Spek, 2009 ▶).

## Supplementary Material

Crystal structure: contains datablocks global, I. DOI: 10.1107/S1600536809046170/ci2960sup1.cif

Structure factors: contains datablocks I. DOI: 10.1107/S1600536809046170/ci2960Isup2.hkl

Additional supplementary materials:  crystallographic information; 3D view; checkCIF report

## Figures and Tables

**Table 1:** Selected bond lengths (Å)

Cu1—O2	1.9169 (7)
Cu1—O1	2.0857 (8)
Cu1—O3	2.2016 (7)

**Table 2:** Hydrogen-bond geometry (Å, °)

*D*—H⋯*A*	*D*—H	H⋯*A*	*D*⋯*A*	*D*—H⋯*A*
O3—H1*O*3⋯O6	0.95	1.61	2.5034 (13)	154
N1—H1*N*1⋯O5^i^	0.86	2.44	3.169 (2)	143
N1—H2*N*1⋯O2^ii^	0.86	2.47	3.0810 (19)	129
N1—H2*N*1⋯O1^iii^	0.86	2.50	3.3243 (18)	161
N2—H1*N*2⋯O7^iv^	0.86	2.06	2.906 (2)	169
N2—H2*N*2⋯O4^iii^	0.86	2.07	2.8811 (14)	157
N3—H1*N*3⋯O6^iv^	0.86	2.02	2.860 (2)	167
N3—H2*N*3⋯O5^i^	0.86	2.12	2.937 (2)	157
N4—H1*N*4⋯O1*W* ^v^	0.86	2.10	2.916 (2)	157
N4—H2*N*4⋯O6^vi^	0.86	2.56	3.0760 (18)	119
N4—H2*N*4⋯O7^i^	0.86	2.26	2.9973 (17)	144
N5—H1*N*5⋯O2^ii^	0.86	2.06	2.8484 (15)	152
N5—H2*N*5⋯O7^i^	0.86	2.03	2.8273 (17)	153
N6—H1*N*6⋯O3^iii^	0.86	2.18	3.0140 (14)	164
N6—H2*N*6⋯O4	0.86	1.99	2.8387 (18)	170
O1*W*—H1*W*1⋯O4^v^	0.78	2.52	3.032 (2)	124
O1*W*—H2*W*1⋯O1^ii^	0.90	2.03	2.932 (2)	175

Symmetry codes: (i) 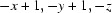; (ii) 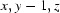; (iii) 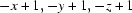; (iv) 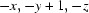; (v) 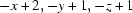; (vi) 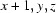.

**Fig. 1.**
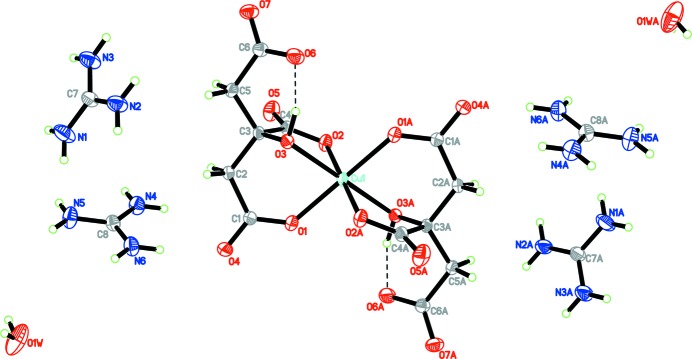
The molecular structure of the title compound with atom labels and 30% probability ellipsoids for non-H atoms. Molecules/atoms with suffix A are generated by the symmetry operation (1-x, 2-y, 1-z). Intramolecular hydrogen bonds are shown as dashed lines.

**Fig. 2.**
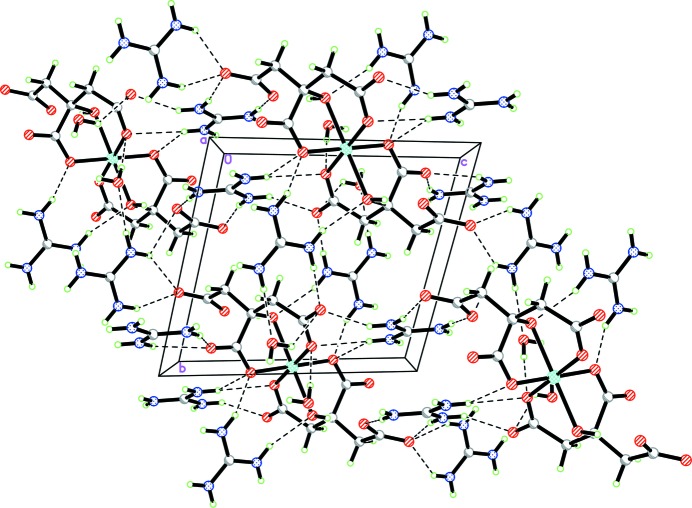
The crystal packing of title compound, viewed down the a axis, showing hydrogen-bonded (dashed lines) three-dimensional framework.

## supplementary crystallographic information

### Comment

Citric acid or 2-hydroxy-1,2,3-propanetricarboxylic acid contains three carboxyl groups. It is an intermediate in the citric acid cycle in living organisms. It can be added to the food and soft drinks to add a sour or an acidic taste. Guanidine can be formed by the oxidation of guanine as a final product of the protein metabolism. The copper(II) ion in this crystal is coordinated to two citrate ions by the oxygen atoms and the four guanidinium ions neutralize the complex charge (Raczyńska *et al.*, 2003; Yamada *et al.*, 2009; Sigman *et al.*, 1993).

The asymmetric unit of title compound contains half of a Cu^II^ complex anion, two guanidinium cations and a water solvent molecule, the other half is symmetry generated [symmetry code: -*x* + 1, -*y* + 2, -*z* + 1] (Fig. 1). The Cu^II^ ion lies on a crystallographic inversion center and is coordinated to six O atoms from two citrate anions to form an octahedral geometry. Four protons are deprotonated from two citric acid molecules to four guanidine molecules resulting in the formation of ions. The geometrical parameters of guanidinium cations agree with those previously reported (Al-Dajani *et al.*, 2009). An intramolecular O3—H1O3···O6 hydrogen bond generates an *S*(6) ring motif (Bernstein *et al.*, 1995).

In crystal structure (Fig. 2), all guanidinium N–H groups participate in the formation of a three-dimensional framework through N—H···O hydrogen bonds (Table 2). The structure are also stabilized by intermolecular O1W—H1W1···O4 and O1W—H2W1···O1 hydrogen bonds.

### Experimental

Citric acid (anhydrous) (0.02 mol, 3.85 g) was dissolved in THF in a flat bottom flask with magnetic stirrer. In a separating funnel, guanidine carbonate (0.02 mol, 3.6 g), 99% [H_2_NC(NH)NH_2_].2H_2_CO_3_ was dissolved in THF. The guanidine solution was added in small portions to the flask of citric acid with stirring. The reaction mixture was refluxed for 1 h. After cooling the reaction mixture to room temperature, CuCl_2_ (0.01 mol, 1.45 g) was added with stirring for 3 h. Blue crystals formed were washed with *N*,*N*-dimethylformamide followed by methanol and dried at 353 K.

### Refinement

O-bound H atoms were located in a difference Fourier map and refined as riding on their parent atom, with *U*_iso_(H) = 1.5*U*_eq_(O). The remaining H atoms were positioned geometrically [C–H = 0.97 Å and N–H = 0.86 Å] and refined using a riding model, with *U*_iso_(H) = 1.2*U*_eq_(C,N).

### Crystal data

**Table:**

(CH_6_N_3_)_4_[Cu(C_6_H_5_O_7_)_2_]·2H_2_O	*Z* = 1
*M**_r_* = 718.12	*F*(000) = 375
Triclinic, *P*1	*D*_x_ = 1.508 Mg m^−^^3^
Hall symbol: -P 1	Mo *K*α radiation, λ = 0.71073 Å
*a* = 9.0426 (1) Å	Cell parameters from 9680 reflections
*b* = 9.7763 (2) Å	θ = 2.3–34.9°
*c* = 10.3366 (2) Å	µ = 0.78 mm^−^^1^
α = 96.503 (1)°	*T* = 296 K
β = 105.441 (1)°	Block, blue
γ = 112.306 (1)°	0.60 × 0.39 × 0.32 mm
*V* = 791.01 (2) Å^3^	

### Data collection

**Table:**

Bruker SMART APEXII CCD area-detector diffractometer	7051 independent reflections
Radiation source: fine-focus sealed tube	6306 reflections with *I* > 2σ(*I*)
graphite	*R*_int_ = 0.024
φ and ω scans	θ_max_ = 35.3°, θ_min_ = 2.1°
Absorption correction: multi-scan (*SADABS*; Bruker, 2005)	*h* = −14→14
*T*_min_ = 0.653, *T*_max_ = 0.787	*k* = −15→15
37237 measured reflections	*l* = −16→16

### Refinement

**Table:**

Refinement on *F*^2^	Primary atom site location: structure-invariant direct methods
Least-squares matrix: full	Secondary atom site location: difference Fourier map
*R*[*F*^2^ > 2σ(*F*^2^)] = 0.031	Hydrogen site location: inferred from neighbouring sites
*wR*(*F*^2^) = 0.094	H-atom parameters constrained
*S* = 1.05	*w* = 1/[σ^2^(*F*_o_^2^) + (0.0533*P*)^2^ + 0.108*P*] where *P* = (*F*_o_^2^ + 2*F*_c_^2^)/3
7051 reflections	(Δ/σ)_max_ < 0.001
206 parameters	Δρ_max_ = 0.44 e Å^−^^3^
0 restraints	Δρ_min_ = −0.49 e Å^−^^3^

### Special details

**Table:**

Geometry. All e.s.d.'s (except the e.s.d. in the dihedral angle between two l.s. planes) are estimated using the full covariance matrix. The cell e.s.d.'s are taken into account individually in the estimation of e.s.d.'s in distances, angles and torsion angles; correlations between e.s.d.'s in cell parameters are only used when they are defined by crystal symmetry. An approximate (isotropic) treatment of cell e.s.d.'s is used for estimating e.s.d.'s involving l.s. planes.
Refinement. Refinement of *F*^2^ against ALL reflections. The weighted *R*-factor *wR* and goodness of fit *S* are based on *F*^2^, conventional *R*-factors *R* are based on *F*, with *F* set to zero for negative *F*^2^. The threshold expression of *F*^2^ > σ(*F*^2^) is used only for calculating *R*-factors(gt) *etc*. and is not relevant to the choice of reflections for refinement. *R*-factors based on *F*^2^ are statistically about twice as large as those based on *F*, and *R*- factors based on ALL data will be even larger.

### Fractional atomic coordinates and isotropic or equivalent isotropic displacement parameters (Å^2^)

**Table:**

	*x*	*y*	*z*	*U*_iso_*/*U*_eq_	
Cu1	0.5000	1.0000	0.5000	0.02544 (5)	
O1	0.66219 (12)	0.89568 (10)	0.55711 (9)	0.04046 (18)	
O2	0.55673 (11)	1.02356 (8)	0.33541 (8)	0.03511 (15)	
O3	0.32813 (9)	0.77094 (8)	0.36675 (7)	0.02790 (12)	
H1O3	0.2276	0.7661	0.3035	0.042*	
O4	0.76260 (15)	0.72314 (13)	0.55564 (10)	0.0536 (3)	
O5	0.57936 (15)	0.90234 (12)	0.15326 (10)	0.0506 (2)	
O7	0.09687 (12)	0.65390 (12)	−0.05629 (9)	0.04401 (19)	
O6	0.10222 (13)	0.74648 (13)	0.15156 (9)	0.0480 (2)	
C1	0.66253 (14)	0.77440 (12)	0.49850 (10)	0.03244 (18)	
C2	0.53925 (14)	0.68374 (11)	0.35373 (10)	0.03180 (18)	
H2A	0.4699	0.5826	0.3601	0.038*	
H2B	0.6052	0.6721	0.2966	0.038*	
C3	0.42059 (12)	0.74833 (10)	0.27880 (9)	0.02586 (14)	
C4	0.52711 (13)	0.90199 (11)	0.25150 (10)	0.02936 (16)	
C5	0.29501 (13)	0.63732 (12)	0.14090 (10)	0.03315 (18)	
H5A	0.3568	0.6313	0.0784	0.040*	
H5B	0.2432	0.5367	0.1567	0.040*	
C6	0.15557 (13)	0.68329 (13)	0.07232 (11)	0.03335 (18)	
N1	0.33925 (19)	0.13097 (19)	0.12541 (16)	0.0627 (4)	
H1N1	0.3944	0.1103	0.0759	0.075*	
H2N1	0.3625	0.1237	0.2099	0.075*	
N2	0.13528 (17)	0.20918 (16)	0.14703 (11)	0.0503 (3)	
H1N2	0.0587	0.2390	0.1118	0.060*	
H2N2	0.1577	0.2022	0.2316	0.060*	
N3	0.18333 (18)	0.18500 (18)	−0.05808 (13)	0.0548 (3)	
H1N3	0.1067	0.2149	−0.0929	0.066*	
H2N3	0.2372	0.1622	−0.1078	0.066*	
C7	0.21821 (16)	0.17412 (15)	0.07130 (13)	0.0409 (2)	
N4	0.86185 (16)	0.53693 (13)	0.28012 (13)	0.0475 (2)	
H1N4	0.8743	0.6260	0.3163	0.057*	
H2N4	0.8867	0.5223	0.2066	0.057*	
N5	0.78586 (17)	0.28597 (12)	0.28065 (14)	0.0506 (3)	
H1N5	0.7486	0.2106	0.3171	0.061*	
H2N5	0.8111	0.2727	0.2071	0.061*	
N6	0.76591 (17)	0.44356 (13)	0.45058 (12)	0.0469 (2)	
H1N6	0.7286	0.3687	0.4875	0.056*	
H2N6	0.7782	0.5324	0.4872	0.056*	
C8	0.80435 (14)	0.42221 (12)	0.33777 (12)	0.03575 (19)	
O1W	0.9973 (2)	0.13741 (17)	0.59833 (19)	0.0955 (6)	
H1W1	0.9976	0.1225	0.5223	0.143*	
H2W1	0.8945	0.0656	0.5902	0.143*	

### Atomic displacement parameters (Å^2^)

**Table:**

	*U*^11^	*U*^22^	*U*^33^	*U*^12^	*U*^13^	*U*^23^
Cu1	0.03201 (8)	0.02210 (7)	0.02568 (7)	0.01325 (6)	0.01258 (6)	0.00553 (5)
O1	0.0452 (4)	0.0371 (4)	0.0360 (4)	0.0230 (3)	0.0056 (3)	−0.0025 (3)
O2	0.0494 (4)	0.0258 (3)	0.0361 (3)	0.0148 (3)	0.0244 (3)	0.0102 (3)
O3	0.0326 (3)	0.0312 (3)	0.0270 (3)	0.0169 (3)	0.0150 (2)	0.0095 (2)
O4	0.0671 (6)	0.0638 (6)	0.0356 (4)	0.0484 (5)	0.0001 (4)	0.0013 (4)
O5	0.0680 (6)	0.0506 (5)	0.0475 (5)	0.0245 (5)	0.0415 (5)	0.0142 (4)
O7	0.0438 (4)	0.0615 (5)	0.0297 (3)	0.0260 (4)	0.0114 (3)	0.0117 (3)
O6	0.0495 (5)	0.0745 (6)	0.0368 (4)	0.0434 (5)	0.0156 (4)	0.0118 (4)
C1	0.0374 (5)	0.0347 (4)	0.0283 (4)	0.0200 (4)	0.0097 (3)	0.0055 (3)
C2	0.0381 (5)	0.0299 (4)	0.0298 (4)	0.0209 (4)	0.0075 (3)	0.0030 (3)
C3	0.0307 (4)	0.0259 (3)	0.0257 (3)	0.0156 (3)	0.0118 (3)	0.0055 (3)
C4	0.0349 (4)	0.0310 (4)	0.0297 (4)	0.0173 (3)	0.0166 (3)	0.0097 (3)
C5	0.0345 (4)	0.0344 (4)	0.0302 (4)	0.0184 (4)	0.0081 (3)	0.0007 (3)
C6	0.0317 (4)	0.0402 (5)	0.0306 (4)	0.0168 (4)	0.0118 (3)	0.0095 (4)
N1	0.0661 (8)	0.0851 (10)	0.0603 (7)	0.0545 (8)	0.0185 (6)	0.0314 (7)
N2	0.0583 (7)	0.0732 (8)	0.0362 (5)	0.0412 (6)	0.0187 (5)	0.0210 (5)
N3	0.0626 (7)	0.0884 (9)	0.0431 (5)	0.0535 (7)	0.0265 (5)	0.0286 (6)
C7	0.0425 (6)	0.0463 (6)	0.0403 (5)	0.0248 (5)	0.0127 (4)	0.0160 (4)
N4	0.0615 (7)	0.0363 (5)	0.0504 (6)	0.0167 (5)	0.0300 (5)	0.0196 (4)
N5	0.0674 (7)	0.0337 (4)	0.0606 (7)	0.0170 (5)	0.0423 (6)	0.0136 (4)
N6	0.0701 (7)	0.0422 (5)	0.0486 (6)	0.0307 (5)	0.0365 (5)	0.0214 (4)
C8	0.0382 (5)	0.0329 (4)	0.0413 (5)	0.0146 (4)	0.0197 (4)	0.0144 (4)
O1W	0.0812 (9)	0.0655 (8)	0.1106 (12)	−0.0041 (7)	0.0578 (9)	−0.0212 (8)

### Geometric parameters (Å, °)

**Table:**

Cu1—O2^i^	1.9169 (7)	C5—H5B	0.97
Cu1—O2	1.9169 (7)	N1—C7	1.3292 (16)
Cu1—O1	2.0857 (8)	N1—H1N1	0.86
Cu1—O1^i^	2.0857 (8)	N1—H2N1	0.86
Cu1—O3^i^	2.2015 (7)	N2—C7	1.3189 (17)
Cu1—O3	2.2016 (7)	N2—H1N2	0.86
O1—C1	1.2704 (12)	N2—H2N2	0.86
O2—C4	1.2798 (12)	N3—C7	1.3162 (16)
O3—C3	1.4401 (11)	N3—H1N3	0.86
O3—H1O3	0.95	N3—H2N3	0.86
O4—C1	1.2432 (13)	N4—C8	1.3255 (14)
O5—C4	1.2286 (12)	N4—H1N4	0.86
O7—C6	1.2464 (13)	N4—H2N4	0.86
O6—C6	1.2678 (13)	N5—C8	1.3232 (15)
C1—C2	1.5261 (14)	N5—H1N5	0.86
C2—C3	1.5273 (13)	N5—H2N5	0.86
C2—H2A	0.97	N6—C8	1.3191 (15)
C2—H2B	0.97	N6—H1N6	0.86
C3—C5	1.5334 (13)	N6—H2N6	0.86
C3—C4	1.5513 (13)	O1W—H1W1	0.78
C5—C6	1.5234 (14)	O1W—H2W1	0.90
C5—H5A	0.97		
			
O2^i^—Cu1—O2	179.999 (1)	O5—C4—C3	119.72 (9)
O2^i^—Cu1—O1	89.36 (4)	O2—C4—C3	116.95 (8)
O2—Cu1—O1	90.64 (4)	C6—C5—C3	113.23 (8)
O2^i^—Cu1—O1^i^	90.64 (4)	C6—C5—H5A	108.9
O2—Cu1—O1^i^	89.36 (4)	C3—C5—H5A	108.9
O1—Cu1—O1^i^	180.00 (3)	C6—C5—H5B	108.9
O2^i^—Cu1—O3^i^	80.58 (3)	C3—C5—H5B	108.9
O2—Cu1—O3^i^	99.42 (3)	H5A—C5—H5B	107.7
O1—Cu1—O3^i^	97.62 (3)	O7—C6—O6	123.57 (10)
O1^i^—Cu1—O3^i^	82.38 (3)	O7—C6—C5	119.46 (10)
O2^i^—Cu1—O3	99.42 (3)	O6—C6—C5	116.94 (9)
O2—Cu1—O3	80.58 (3)	C7—N1—H1N1	120.0
O1—Cu1—O3	82.38 (3)	C7—N1—H2N1	120.0
O1^i^—Cu1—O3	97.62 (3)	H1N1—N1—H2N1	120.0
O3^i^—Cu1—O3	180.0	C7—N2—H1N2	120.0
C1—O1—Cu1	131.70 (7)	C7—N2—H2N2	120.0
C4—O2—Cu1	116.90 (6)	H1N2—N2—H2N2	120.0
C3—O3—Cu1	102.63 (5)	C7—N3—H1N3	120.0
C3—O3—H1O3	103.2	C7—N3—H2N3	120.0
Cu1—O3—H1O3	113.8	H1N3—N3—H2N3	120.0
O4—C1—O1	122.02 (10)	N3—C7—N2	119.75 (11)
O4—C1—C2	116.47 (9)	N3—C7—N1	119.69 (13)
O1—C1—C2	121.51 (9)	N2—C7—N1	120.54 (12)
C1—C2—C3	117.43 (7)	C8—N4—H1N4	120.0
C1—C2—H2A	107.9	C8—N4—H2N4	120.0
C3—C2—H2A	107.9	H1N4—N4—H2N4	120.0
C1—C2—H2B	107.9	C8—N5—H1N5	120.0
C3—C2—H2B	107.9	C8—N5—H2N5	120.0
H2A—C2—H2B	107.2	H1N5—N5—H2N5	120.0
O3—C3—C2	107.59 (7)	C8—N6—H1N6	120.0
O3—C3—C5	109.31 (8)	C8—N6—H2N6	120.0
C2—C3—C5	110.83 (7)	H1N6—N6—H2N6	120.0
O3—C3—C4	110.36 (7)	N6—C8—N5	120.42 (10)
C2—C3—C4	109.34 (8)	N6—C8—N4	120.42 (11)
C5—C3—C4	109.39 (8)	N5—C8—N4	119.15 (11)
O5—C4—O2	123.33 (10)	H1W1—O1W—H2W1	101.6
			
O2^i^—Cu1—O1—C1	118.93 (11)	Cu1—O3—C3—C4	−32.73 (8)
O2—Cu1—O1—C1	−61.07 (11)	C1—C2—C3—O3	−55.52 (11)
O3^i^—Cu1—O1—C1	−160.66 (11)	C1—C2—C3—C5	−174.99 (9)
O3—Cu1—O1—C1	19.33 (11)	C1—C2—C3—C4	64.35 (11)
O1—Cu1—O2—C4	58.64 (8)	Cu1—O2—C4—O5	−169.11 (10)
O1^i^—Cu1—O2—C4	−121.36 (8)	Cu1—O2—C4—C3	10.43 (12)
O3^i^—Cu1—O2—C4	156.47 (8)	O3—C3—C4—O5	−161.50 (10)
O3—Cu1—O2—C4	−23.53 (8)	C2—C3—C4—O5	80.34 (12)
O2^i^—Cu1—O3—C3	−149.08 (5)	C5—C3—C4—O5	−41.19 (13)
O2—Cu1—O3—C3	30.92 (5)	O3—C3—C4—O2	18.95 (12)
O1—Cu1—O3—C3	−61.01 (5)	C2—C3—C4—O2	−99.21 (10)
O1^i^—Cu1—O3—C3	118.99 (5)	C5—C3—C4—O2	139.26 (9)
Cu1—O1—C1—O4	−173.35 (10)	O3—C3—C5—C6	52.33 (11)
Cu1—O1—C1—C2	6.55 (17)	C2—C3—C5—C6	170.76 (9)
O4—C1—C2—C3	−177.27 (11)	C4—C3—C5—C6	−68.62 (10)
O1—C1—C2—C3	2.83 (16)	C3—C5—C6—O7	147.04 (11)
Cu1—O3—C3—C2	86.49 (7)	C3—C5—C6—O6	−34.97 (14)
Cu1—O3—C3—C5	−153.08 (6)		

Symmetry codes: (i) −*x*+1, −*y*+2, −*z*+1.

### Hydrogen-bond geometry (Å, °)

**Table:**

*D*—H···*A*	*D*—H	H···*A*	*D*···*A*	*D*—H···*A*
O3—H1O3···O6	0.95	1.61	2.5034 (13)	154
N1—H1N1···O5^ii^	0.86	2.44	3.169 (2)	143
N1—H2N1···O2^iii^	0.86	2.47	3.0810 (19)	129
N1—H2N1···O1^iv^	0.86	2.50	3.3243 (18)	161
N2—H1N2···O7^v^	0.86	2.06	2.906 (2)	169
N2—H2N2···O4^iv^	0.86	2.07	2.8811 (14)	157
N3—H1N3···O6^v^	0.86	2.02	2.860 (2)	167
N3—H2N3···O5^ii^	0.86	2.12	2.937 (2)	157
N4—H1N4···O1W^vi^	0.86	2.10	2.916 (2)	157
N4—H2N4···O6^vii^	0.86	2.56	3.0760 (18)	119
N4—H2N4···O7^ii^	0.86	2.26	2.9973 (17)	144
N5—H1N5···O2^iii^	0.86	2.06	2.8484 (15)	152
N5—H2N5···O7^ii^	0.86	2.03	2.8273 (17)	153
N6—H1N6···O3^iv^	0.86	2.18	3.0140 (14)	164
N6—H2N6···O4	0.86	1.99	2.8387 (18)	170
O1W—H1W1···O4^vi^	0.78	2.52	3.032 (2)	124
O1W—H2W1···O1^iii^	0.90	2.03	2.932 (2)	175

Symmetry codes: (ii) −*x*+1, −*y*+1, −*z*; (iii) *x*, *y*−1, *z*; (iv) −*x*+1, −*y*+1, −*z*+1; (v) −*x*, −*y*+1, −*z*; (vi) −*x*+2, −*y*+1, −*z*+1; (vii) *x*+1, *y*, *z*.
